# Decay Kinetics of an Interferon Gamma Release Assay with Anti-Tuberculosis Therapy in Newly Diagnosed Tuberculosis Cases

**DOI:** 10.1371/journal.pone.0012502

**Published:** 2010-09-01

**Authors:** Ifedayo M. O. Adetifa, Martin O. C. Ota, Brigitte Walther, Abdulrahman S. Hammond, Moses D. Lugos, David J. Jeffries, Simon A. Donkor, Richard A. Adegbola, Philip C. Hill

**Affiliations:** 1 Bacterial Diseases Programme, Medical Research Council (United Kingdom) Laboratories, Fajara, The Gambia; 2 Statistics and Data Support Unit, Medical Research Council (United Kingdom) Laboratories, Fajara, The Gambia; University of Cape Town, South Africa

## Abstract

**Background:**

Qualitative and quantitative changes in IGRA response offer promise as biomarkers to monitor Tuberculosis (TB) drug therapy, and for the comparison of new interventions. We studied the decay kinetics of TB-specific antigen T-cell responses measured with an in-house ELISPOT assay during the course of therapy.

**Methods:**

Newly diagnosed sputum smear positive TB cases with typical TB chest radiographs were recruited. All patients were given standard anti-TB treatment. Each subject was followed up for 6 months and treatment outcomes were documented. Blood samples were obtained for the ESAT-6 and CFP-10 (EC) ELISPOT at diagnosis, 1-, 2-, 4- and 6-months. Qualitative and quantitative reversion of the ELISPOT results were assessed with McNemar test, conditional logistic regression and mixed-effects hierarchical Poisson models.

**Results:**

A total of 116 cases were recruited and EC ELISPOT was positive for 87% (95 of 109) at recruitment. There was a significant decrease in the proportion of EC ELISPOT positive cases over the treatment period (p<0.001). Most of the reversion occurred between the start and first month of treatment and at completion at 6 months. ESAT-6 had higher median counts compared to CFP-10 at all time points. Counts for each antigen declined significantly with therapy (p<0.001). Reverters had lower median SFUs at the start of treatment compared to non-Reverters for both antigens. Apart from the higher median counts for non-Reverters, no other risk factors for non-reversion were found.

**Conclusions:**

TB treatment induces qualitative and quantitative reversion of a positive in-house IGRA in newly diagnosed cases of active TB disease. As this does not occur reliably in the majority of cured individuals, qualitative and quantitative reversion of an IGRA ELISPOT has limited clinical utility as a surrogate marker of treatment efficacy.

## Introduction

Tuberculosis (TB) is responsible for the greatest number of deaths attributable to a single infectious agent worldwide and most of this mortality is in developing countries.[1] New diagnostic tools, and enhanced treatment strategies, are needed to help combat the TB epidemic. These need to be shown to be useful in TB-endemic tropical settings. Interferon gamma release assays (IGRAs) are utilised in the diagnosis of latent tuberculosis infection (LTBI).[2], [3] However, their performance appears to vary between high burden, resource limited settings and low burden countries.[4] They may also have value in the evaluation of new tools against TB.

The magnitude of the *Mycobacterium (M.) tuberculosis* antigen-specific interferon gamma (IFN-g) T-cell response to infecting *M. tuberculosis* is hypothesized to be proportional to the antigenic load of the infecting organism in human and animal models.[5], [6] We have shown that the quantitative response, as measured by ELISPOT, correlates with exposure to a TB case, reflecting the infectious load of *M. tuberculosis*. Sharply rising counts following exposure may be predictive of TB disease.[7], [8] Effective treatment for LTBI is associated with a decrease in the measured response to ESAT-6 and CFP-10 antigens.[9], [10] Studies have also shown significant qualitative and quantitative reversion of the response following treatment of adult and paediatric TB cases in different settings.[11], [12], [13]

Qualitative and quantitative changes in IGRA response offer promise as biomarkers to monitor the response to TB drug therapy, and for the comparison of new interventions. However, more needs to be known about the nature of the changes in the IGRA response. Having determined that, even in a TB-endemic tropical setting, ELISPOT counts decrease significantly in TB cases after treatment[14], we studied the decay kinetics of TB-specific antigen T-cell responses during the course of treatment.

## Methods

### Participants

Newly diagnosed TB cases at the Medical Research Council (MRC) out-patient clinic and the major government TB chest clinic were consecutively recruited to the study. They were eligible if they were ≥15 years old, were sputum smear positive and had a typical TB chest x-ray. Blood samples were obtained for full blood count, HIV testing and EC ELISPOT at recruitment. In addition, follow-up samples were collected at 1-, 2-, 4- and 6-months of treatment. All patients received standard anti-tuberculosis treatment provided free by the Gambian National TB Control Programme. Patients' clinical outcomes were reviewed at each follow-up visit and their treatment cards checked for compliance/adherence to treatment. Treatment outcomes were documented as cured, completed treatment, defaulted, treatment failure, transferred out and died according to standard World Health Organization (WHO) definitions.[15] Each subject was followed for total period of 6 months. At the end of the follow-up period we documented if the patient was alive/dead, the presence of clinical symptoms suggestive of TB (chronic cough, weight loss, night sweats, etc), a new diagnosis of TB, ongoing re-treatment or treatment for mono- or multi-drug resistant TB.

### Ethics Statement

The joint MRC-Gambian Government Ethics committee gave ethical approval for this study. Written informed consent was obtained from all enrolled study participants.

### Laboratory Procedures

Sputum smear microscopy was done on samples stained with auramine phenol and Ziehl-Neelsen. Isolation and identification of *M. tuberculosis* was as previously described using Lowenstein-Jensen medium and BACTEC 9000 liquid media.[16] HIV status was determined by enzyme linked immunosorbent assays (Murex 1.2.0, Abbott-Murex Biotec, Dartford, Kent, UK), Hexagon HIV (Human Diagnostics GmbH, Wiesbaden, Germany) and type specific immunoblotting kit (Pepti-LAV I/II, BIORAD, Marnes-la-Coquette, France) for confirmation.

The *ex-vivo* ELISPOT assays for IFN-γ were performed as previously described.[17], [18] The antigens used in this study were 15 amino acids long synthetic, sequential peptides spanning the length of ESAT-6 and CFP-10 (ABC, Imperial College, London, UK). Each peptide overlapped its adjacent peptide by 10 residues. ESAT-6 and CFP-10 peptide pools were used at 5μg/ml while Phytohaemaglutinin (PHA 5 μg/ml; Sigma, Aldrich, UK) was used as positive control with medium as negative control.

Peripheral blood mononuclear cell (PBMCs) separation and fresh ex vivo ELISPOT were done within 4 hours of sampling by laboratory staff blind to clinical information: 200,000 PBMCs, per well were incubated with all antigens and tested in duplicate wells. Spot forming units (SFUs) were counted with an automated ELISPOT reader (AID-GmbH, Strasberg, Germany). Positive test wells were pre-defined as containing at least 8 SFUs/well/2×10^5^ PBMCs more than negative control wells.[19] For a positive ESAT-6/CFP-10 result it was necessary for one or more pools of overlapping peptides to be positive. PHA wells were set to at least 150 SFUs/well/2×10^5^ PBMCs above negative control wells. Negative control wells were required to have less than 20 SFUs/well/2×10^5^ PBMCs.[19] The ELISPOT was considered to have failed if the specifications for the negative control and PHA wells were not met. With respect to reproducibility, we have previously shown that within-subject variability of our ELISPOT assay is very small compared to between-subject variability.[14]

### Data management and analysis

All data were double entered into an Access relational database. Statistical analyses were performed using Stata 11.1 (Stata Corp LP, College Station, Texas). The main outcomes of interest were qualitative and quantitative reversion of the EC ELISPOT during the period TB treatment. The impact of HIV status, age, sputum smear grade and findings on chest radiographs on reversion was also evaluated. McNemar tests were used to assess the relationship between qualitative CFP-10, ESAT-6, final EC ELISPOT and time points at testing. Conditional logistic regression was used to test for trend across time points. Quantitative reversion was assessed by Wilcoxon signed-rank test and mixed-effect hierarchical Poisson models adjusting for possible confounding factors, such as age, gender, HIV status, BCG scar, sputum smear grade and severity of radiologic disease. A p-value <0.05 was considered significant.

## Results

### Study population

One hundred and sixteen smear positive TB cases were eligible; 113 (97%,) were culture positive. The median age was 27.5 years (minimum: 15 years, maximum: 71 years), there were 3 times more males than females and less than 10% were HIV infected (Table 1). Ninety-six (82.8%) cases completed treatment in 6 months, 4 (3.4%) defaulted, 1(0.9%) died, 11(9.5%) transferred out and for 4 (3.4%) treatment outcome could not be determined. There were 112 with 2 or more EC ELISPOT results and 86 with 3 or more results.

**Table 1:** Clinical and demographic characteristics of study population.

Characteristic	All cases (n = 116)
**Age, years**	
Median (IQR)	27.5 (22–36)
**Sex**	
Female	31 (26.7)
Male	85 (73.3)
**Ethnic group**	
Mandinka	46 (39.7)
Jola	27 (23.3)
Wolof	14 (12.1)
Fula	7 (6.0)
Others	22 (19.0)
**Sputum smear grade**	
Low	2 (1.7)
Medium	25 (21.6)
High	89 (76.7)
**BCG scar**	
Present	39 (33.6)
Absent	61 (52.6)
Unknown	16 (13.8)
**HIV status**	
Positive	10 (8.6)
Negative	101 (87.1)
Unknown	5 (4.3)

### Qualitative results over time

**Figure 1:**
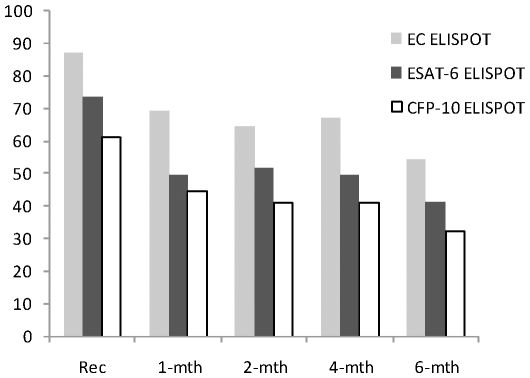
Changes in proportions of tuberculosis cases positive for combined and individual antigen ELISPOT assay during TB treatment and follow-up.

At recruitment, 95 of 109 cases (87.2%; 95% confidence interval (95% CI): 79.4–92.8%) with valid results had a positive EC ELISPOT. As seen in Figure 1, there was a significant change in the proportion EC ELISPOT positive over the time of treatment (coefficient (coeff): −0.38, 95% CI: −0.54 to −0.22, p<0.001). Most of the qualitative reversion seen occurred between recruitment and first month of therapy (87.2% vs. 70.0% (95% CI: 56.8–81.2%) positive, p<0.001, McNemar's test) and at completion of treatment at 6 months (87.2% vs. 54.3% (95% CI: 41.9–66.3%) positive p<0.001, McNemar's test).

Four (4.7%) cases did not achieve sputum conversion to negative at 2 months. Their EC ELISPOT remained positive for the entire course of therapy, although they were declared cured by completion of the standard treatment regimen. Of the cases with ELISPOT results at 6 months, only 1 (14.3%) out of 7 with negative results at recruitment had conversion to a positive result. This subject's sputum had the maximum smear grade at diagnosis, the TB affected 3 lung zones and was one of those for whom treatment outcome could not be determined.

#### ESAT-6

There were 85 cases (73.3%; 95% CI: 65.1–81.4%) with positive ESAT-6 results. Following 1 and 6 months of treatment, 49.2% (95%CI: 36.5–61.9%) and 41.0% (95%CI: 29.9–52.2) remained positive respectively. Overall, there was 24% reversion at 1-month and 33% reversion at 6-months. The change in proportion of positive ESAT-6 results decreased significantly from recruitment to the end of the first month of treatment (p = 0.028, McNemar test) and between recruitment and 6 months of therapy (p<0.001, McNemar test). Overall the positivity rate changed significantly with duration of treatment (coeff: −0.35, 95% CI: −0.48 to −0.21, p<0.001). Only 1(5.2%) out of 19 cases with a negative result at recruitment for whom 6-month results were available (78) became positive. This subject had a sputum smear grade of 2+, 3 lung zones affected by disease without cavities on chest radiograph and was declared cured at the end of TB therapy.

#### CFP-10

There were 70 cases (62.0%; 95% CI: 52.3–70.9%) positive for CFP-10 at recruitment. This declined to 44.4% (95%CI: 31.9–57.1%) after one month of anti-tuberculosis treatment and to 32.1% (95%CI: 21.5–42.6%) after 6 months of treatment. The reversion of test results at the 1^st^ and 6^th^ months of therapy compared to baseline were statistically significant (p = 0.023 and p<0.001 at 1 and 6 months, respectively). Across follow-up periods and with treatment, the change in positivity was also significant (coeff.: −0.23, 95% CI: −0.35 to −0.12, p<0.001). CFP-10 results were available at 6 months for 78 cases. Five (16.8%) of those with negative results at recruitment (30) became CFP-10 positive after completion of TB therapy. All 5 had the maximum smear grade on sputum microscopy at diagnosis, 3–4 lung zones affected by TB and 4 of 5 were cured at 6 months. The other did not have adequate treatment outcome data.

The difference in proportions of reverters for both antigens was not significant (ESAT-6: 28/71 (58.3%), CFP-10: 28/85 (47.5%), p = 0.26).

### Quantitative results with treatment over time

#### ESAT-6

**Figure 2:**
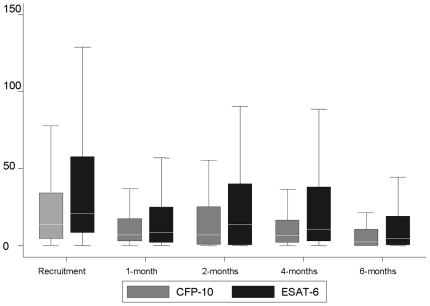
Comparison of median SFUs across time points for ESAT-6 and CFP-10. Error bars represent the interquartile range (IQR).

The median decline in SFUs was 1.7 and 5.5-fold from recruitment to the end of the 1^st^ and 6^th^ month of treatment, respectively (Figure 2). The median SFUs at the start of treatment was 20.8 [interquartile range (IQR): 8.5–57.8], dropping to 8.5 [IQR 2.0–25.0] at 1-month and 4.5 [IQR 0.5–19.0] on completion of treatment. Similar to the qualitative results, ESAT-6 SFUs showed a 2-step decline in SFUs within the first month of therapy (p = 0.06) and between the 1st and 6^th^ month of treatment (p<0.001). Overall, there was a significant decline in SFUs over time with increasing duration of treatment (p<0.0001). The effect of treatment and time on ESAT-6 SFUs remained significant after adjusting for age, gender, ethnicity, HIV status, BCG scar status, sputum grade, and severity of radiologic disease.

#### CFP-10

Median values of CFP-10 were significantly lower than those for ESAT-6 SFUs at diagnosis (p = 0.002) and all other time points but after the 1st month of treatment (p = 0.16, p = 0.029, p = 0.006, and 0.06, respectively). There was a 2-fold median decline in CFP-10 SFUs between recruitment and the first month of treatment increasing to a 4.5-fold median decline between recruitment and 6th months of treatment. The declines in median SFUs between recruitment (13.5, [IQR 4.5–34.3]) and 1-month (7.0, [IQR 3.0–17.5]) and between recruitment and 6-months, were statistically significant (p = 0.03 and p<0.001 respectively). Overall, there was a significant decline in SFUs over time with increasing duration of treatment (p<0.001). This was unaffected by sputum grade, gender, severity of radiologic disease, BCG scar status and ethnicity.

### Quantitative ELISPOT results in Reverters compared to non-Reverters

**Figure 3:**
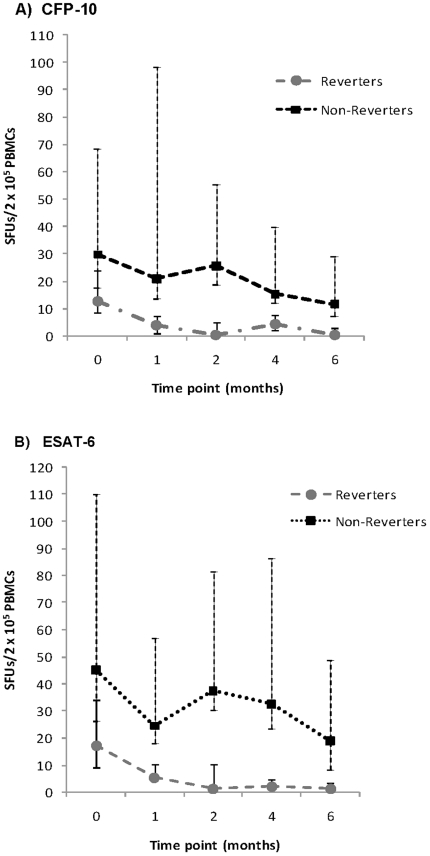
Comparison of median SFUs across time points in Reverters and non-Reverters. A) CFP-10, B) ESAT-6. Error bars represent the interquartile range (IQR).

A group of cases with positive results at onset of treatment and negative results after 6 months of therapy i.e. Reverters were compared to non-Reverters. For ESAT-6, there was approximately a median 15.2-fold change in SFUs from start of to completion of treatment for Reverters and a 2-fold change for non-Reverters. Similarly, there was a 17.4- and 3.3-fold change for CFP-10 for the 2 groups respectively over the same periods. As shown in Figure 3A and 3B, the median ESAT-6 in Reverters at the start and end of treatment was 19.5 [IQR 8.5–36.0] SFUs and 1.5 [IQR 0.0–3.5] SFUs compared to 44 [IQR 19.0–120.0] SFUs and 19.0 [IQR 11.5–54.5] SFUs respectively in non-Reverters. For CFP-10, median SFUs at the start and completion of treatment were 13.0 [IQR 4.5–24.0] SFUs and 0.5 [IQR 0.0–3.0] SFUs respectively for Reverters compared to 35.5 [IQR 21.5–68.5] and SFUs 14.5 [IQR 6.5–34.0] for non-Reverters. Apart from these higher median counts at onset of treatment, no other risk factors for non-reversion were found. For ESAT-6, 28 of 59 (47.5%) reverted on completion of therapy compared to 58.3% (28 of 48) for CFP-10. However, the difference in proportions of Reverters for both antigens was not significant (p = 0.26).

For ESAT-6 and CFP-10, 15 and 21 cases respectively experienced reversion and then at least one conversion. No risk factors for this phenomenon were identified and the SFUs at start of treatment, during the reversion and conversion episodes were not closer to the cut-off for positive results than other episodes.

## Discussion

This study describes the decay kinetics of an in-house TB specific ELISPOT test with antituberculosis treatment in newly diagnosed TB cases in a high burden setting. We found approximately 17.2% of cases had qualitative reversion within the 1st month of treatment with an increase to 32.9% by 6 months. Our data show a 2-step decline in qualitative and quantitative test results: the 1st and 6th month of follow-up showed the most significant change in ELISPOT count. Comparison of quantitative responses between reverters and non-reverters showed that non-reverters started at a much higher level, with some differences seen according to the antigen assessed. These results provide a complete picture of ELISPOT decay kinetics in TB cases and provide insights into the usefulness of the readout as a Biomarker in TB research and practice.

The only factor associated with non-reversion was the presence of higher SFUs for each antigen at baseline and throughout the treatment follow-up period. Interestingly, the decline in median SFUs for both antigens were similar for the reverter and non-reverter groups. It appears that there is a threshold for the amount of decline possible in the number of sensitised effector cells (SFUs) per unit time with treatment, depending on the initial result. Interestingly, when compared to contacts in the community using the same test, the median SFUs for reverters decline with treatment to values below those seen in healthy, but latently infected TB contacts. [17], [20]

Our findings of qualitative and quantitative reversion of the EC ELISPOT test are in agreement with our data previously reported by us and other authors for both commercial and in-house IGRAs.[12], [13], [14], [21]–[26] The 2-step reversion we found had been previously described for contacts and cases given antituberculosis drugs as part of regimens for prophylaxis and treatment in other settings.[9] Reports of initial increases in median antigen SFUs following treatment before a decline over period of therapy are not supported by our study.[27], [28] Neither do our data support a report of an increase in median CFP-10 SFUs following completion of treatment after an initial 3-fold decline.[23]

Higher median ESAT-6 SFUs than those of CFP-10 found here is consistent with similar findings in TB disease and latent TB infection in The Gambia.[17] That the reversion followed the same pattern across time points is comparable to findings reported by others. [24], [29] Dominguez et al, found higher median SFUs for CFP-10 at the start of and throughout the treatment period in their study compared to ESAT-6. [23] Also reversion just for CFP-10 and not with ESAT-6 has been reported following treatment in latent *M. tuberculosis* infection.[10], [30] The presence of a significant minority of individuals with EC-ELISPOT conversion after an initial reversion has also been reported by others.[22], [23], [25]

EC-ELISPOT measures the frequencies of effector T-cells sensitised to ESAT-6 and CFP-10 antigens. These cells are thought to closely correlate with antigen load, driven by the burden of *M.tuberculosis*.[5], [6], [31], [32] Therefore it is not surprising that we found significant qualitative and quantitative test reversion with effective therapy. However, 54.3% of our cases remained EC-ELISPOT positive after TB treatment. The short incubation times for the ELISPOT test are aimed at measurement of interferon gamma produced by short lived effector T-cells. However, it is possible that some memory T-cells come into play despite the short incubation period. Furthermore, it has been shown that in some people effector cells continue to circulate in the absence of antigen.[33] Persistent T-cell responses despite clinical and bacteriologically confirmed resolution of active TB or latent TB infection may be due to host, pathogen or environmental components.[10], [13], [22], [34], [35] An alternative explanation is that the mycobacterial burden is simply reduced by effective therapy, rather than eliminated. This may be the case as there is evidence of recurrence of TB of the same molecular fingerprint in treated cases.[36], [37]

The immediate consequence of the ‘high proportion of non-reversion’ of a T-cell response, and significant interindividual differences, is the limited utility of qualitative and quantitative reversion of EC ELISPOT as a marker of treatment efficacy. It is also clearly not a reliable predictor of treatment failure/adverse outcomes. Furthermore, conversion to a positive test in a small number of cases with negative results at the start of treatment was also unrelated to the usual surrogates of disease severity (smear grade, cavitatory radiologic disease) and not predictive of adverse outcomes (clinical and bacteriologic).

Our study was conducted with an in-house ELISPOT test and not the now commercially available versions. However, our in-house test results are similar to results with the commercial test in The Gamiba.[14], [17], [38], [39], [40] No mycobacterial cultures were done for confirmation of sputum conversion at 2 months and to declare a cured status after 6 months of therapy. Our TB cases were treated in the government chest clinics according to national TB guidelines. In addition, we did not have results from all cases at all time points. However, about three-quarters of the study population had samples taken at 3 or more timepoints.

### Conclusions

We found TB treatment induces qualitative and quantitative reversion of a positive IGRA in newly diagnosed cases of active TB disease that occurs in a 2-step manner: there is a significant decline within the first month of treatment and then on completion. However, qualitative and quantitative reversion of a IGRA ELISPOT has limited clinical utility as a surrogate marker of treatment efficacy as it does not occur reliably in the majority of cured individuals.
